# Activation of endogenous arginine vasopressin neurons inhibit food intake: by using a novel transgenic rat line with DREADDs system

**DOI:** 10.1038/s41598-017-16049-2

**Published:** 2017-11-16

**Authors:** Mitsuhiro Yoshimura, Kazuaki Nishimura, Haruki Nishimura, Satomi Sonoda, Hiromichi Ueno, Yasuhito Motojima, Reiko Saito, Takashi Maruyama, Yuki Nonaka, Yoichi Ueta

**Affiliations:** 1 Department of Physiology, School of Medicine, University of Occupational and Environmental Health, Kitakyushu, 807-8555 Japan; 2 Orthopaedic Surgery, School of Medicine, University of Occupational and Environmental Health, Kitakyushu, 807-8555 Japan

## Abstract

Various studies contributed to discover novel mechanisms of central arginine vasopressin (AVP) system responsible for the behaviour albeit endogenous vasopressin activation. We established a novel transgenic rat line which expresses both human muscarinic acetylcholine receptors (hM3Dq), of which ligand is clozapine-N-oxide (CNO), and mCherry fluorescence specifically in AVP neurons. The mCherry neurons that indicate the expression of the hM3Dq gene were observed in the suprachiasmatic (SCN), supraoptic (SON), and paraventricular nuclei (PVN). hM3Dq-mCherry fluorescence was localized mainly in the membrane of the neurons. The mCherry neurons were co-localized with AVP-like immunoreactive (LI) neurons, but not with oxytocin-LI neurons. The induction of Fos, which is the indicator for neuronal activity, was observed in approximately 90% of the AVP-LI neurons in the SON and PVN 90 min after intraperitoneal (i.p.) administration of CNO. Plasma AVP was significantly increased and food intake, water intake, and urine volume were significantly attenuated after i.p. administration of CNO. Although the detailed mechanism has unveiled, we demonstrated, for the first time, that activation of endogenous AVP neurons decreased food intake. This novel transgenic rat line may provide a revolutionary insight into the neuronal mechanism regarding central AVP system responsible for various kind of behaviours.

## Introduction

Development of chemogenetics as well as optogenetics has been provided innovative knowledge in the field of neuroscience^1,2^. In recent research projects, these techniques have been widely used to understand the linkage between central nervous activity and diverse behaviours. Designer receptors exclusively activated by designer drugs (DREADDs) are the most common G-protein coupled receptors (GPCRs) used in chemogenetics^3^. Human muscarinic acetylcholine receptor (hM3Dq), of which ligand is Clozapine-N-oxide (CNO), is one of the pharmacologically modulated GPCRs which enables us to exploit Gq signaling pathway.

In the studies of neurohypophysial hormones (arginine vasopressin (AVP) and oxytocin (OXT)), several ambitious studies based on viral transfection of the DREADDs have been performed on AVP and OXT neurons and their axon terminals^4–7^, however, to our knowledge, there is so far no successful chemogenetic study on all of the AVP neurons based on transgenic approaches. We succeeded in generating a novel transgenic rat line which express AVP-hM3Dq-mCherry fluorescent protein fusion gene in the magnocellular neurosecretory cells (MNCs) of the supraoptic (SON) and the paraventricular (PVN) nuclei. The MNCs in the SON and the PVN synthesize AVP. They project their axon terminals to the posterior pituitary and secrete AVP into the systemic circulation with action potential-dependent regulation^8^. Plasma AVP acts on the kidney as an anti-diuretic hormone via V2 receptor, whilst somato-dendritic released AVP from the MNCs mediates various central actions of AVP^9^.

In this manuscript, we would like to report a successful establishment of an AVP-hM3Dq-mCherry transgenic rat line. In addition, we examined whether the activation of endogenous AVP neurons, by intraperitoneally administered CNO, could affect feeding behavior, by using this transgenic rat line.

## Results

### Establishment of AVP-hM3Dq-mCherry transgenic rat line

**Figure 1:**
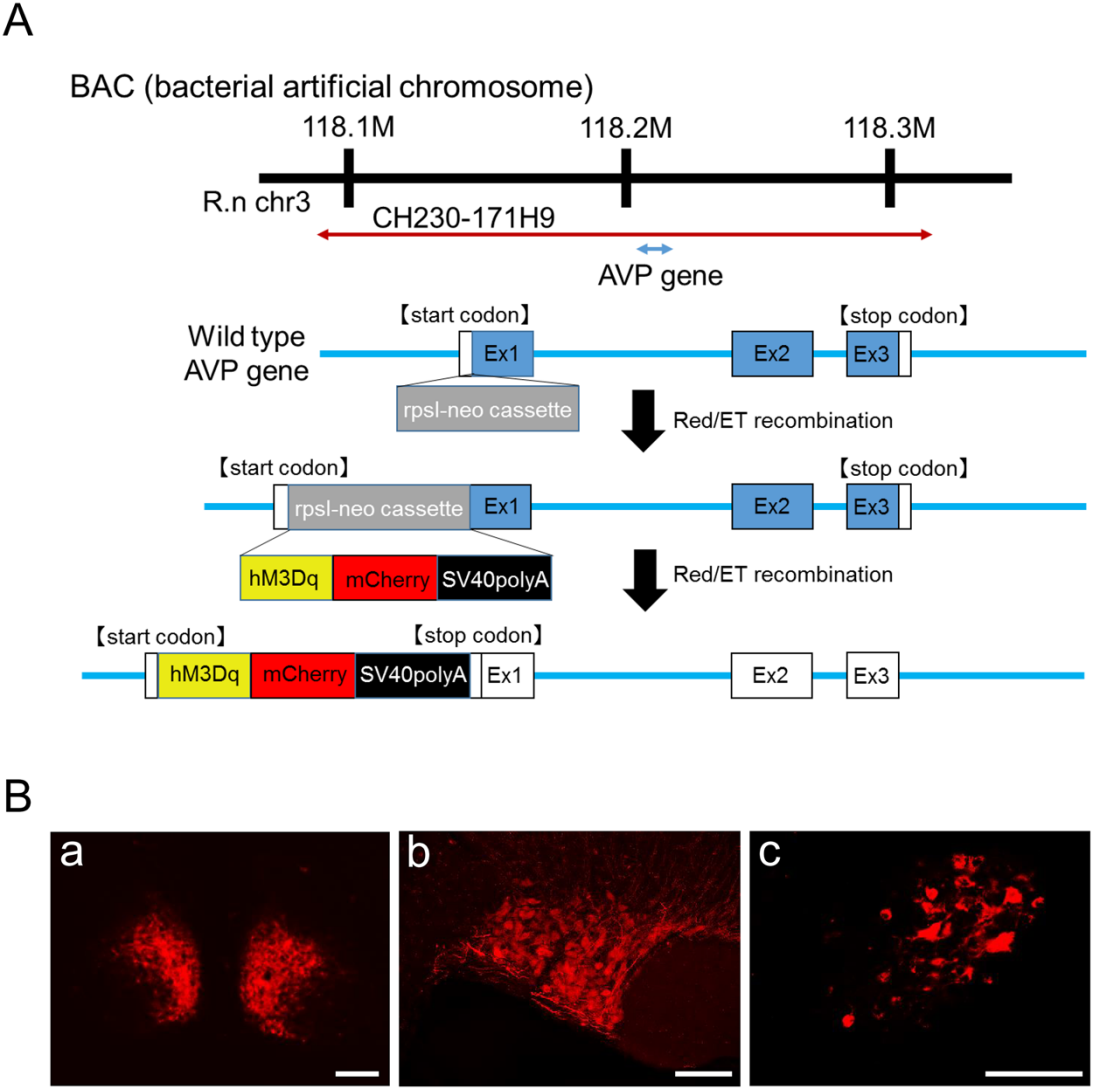
Construction strategy and establishment of an AVP-hM3Dq-mCherry transgenic rat line. (**A**) A chimeric AVP-hM3Dq-mCherry BAC clone transgene construct was purified for microinjections. The hM3Dq-mCherry sequence from the hM3Dq-mCherry cassette (Plasmid #44361, Addgene, Cambridge, MA, USA) was used for the transgene. Then, SV40 poly A sequence was framed to the hM3Dq-mCherry sequence. Finally, thishM3Dq-mCherry-SV40 poly A cassette was introduced into the rat AVP gene in place of the genomic start codon. Transgenic founders and germ line transmission of the BAC transgene construct was assessed by Southern blotting, using ^32^P-labeled mCherry probes. (**B**) The mCherry fluorescent positive neurons were observed in the suprachiasmatic nucleus (SCN, a), supraoptic nucleus (SON, b), and magnocellular division of the paraventricular nucleus (PVN, c). Scale bars indicate 100 μm.

We constructed a chimeric AVP-hM3Dq-mCherry BAC clone transgene (Fig. 1A). hM3Dq-mCherry was specifically expressed under the AVP promoter in the transgenic rat brain. Three transgenic founder male rats were identified by Southern blot analysis using genomic tail DNA with a ^32^P-labeled mCherry probe. The copy number of the transgene was between 10 and 30. All founders were bred and F1 rats were screened by PCR analysis of genomic DNA extracted from ear skin. Although, mCherry was observed in each line, one line (30 copies) which has constant expression of the vivid mCherry in the SON and PVN was determined as an established line.

Vivid mCherry fluorescence was observed in the suprachiasmatic nucleus (SCN) (Fig. 1Ba), SON (Fig. 1Bb), and magnocellular division of the PVN (Fig. 1Bc), where AVP neurons are known to be localised.

### Specificity of hM3Dq-mCherry in AVP neurons

**Figure 2:**
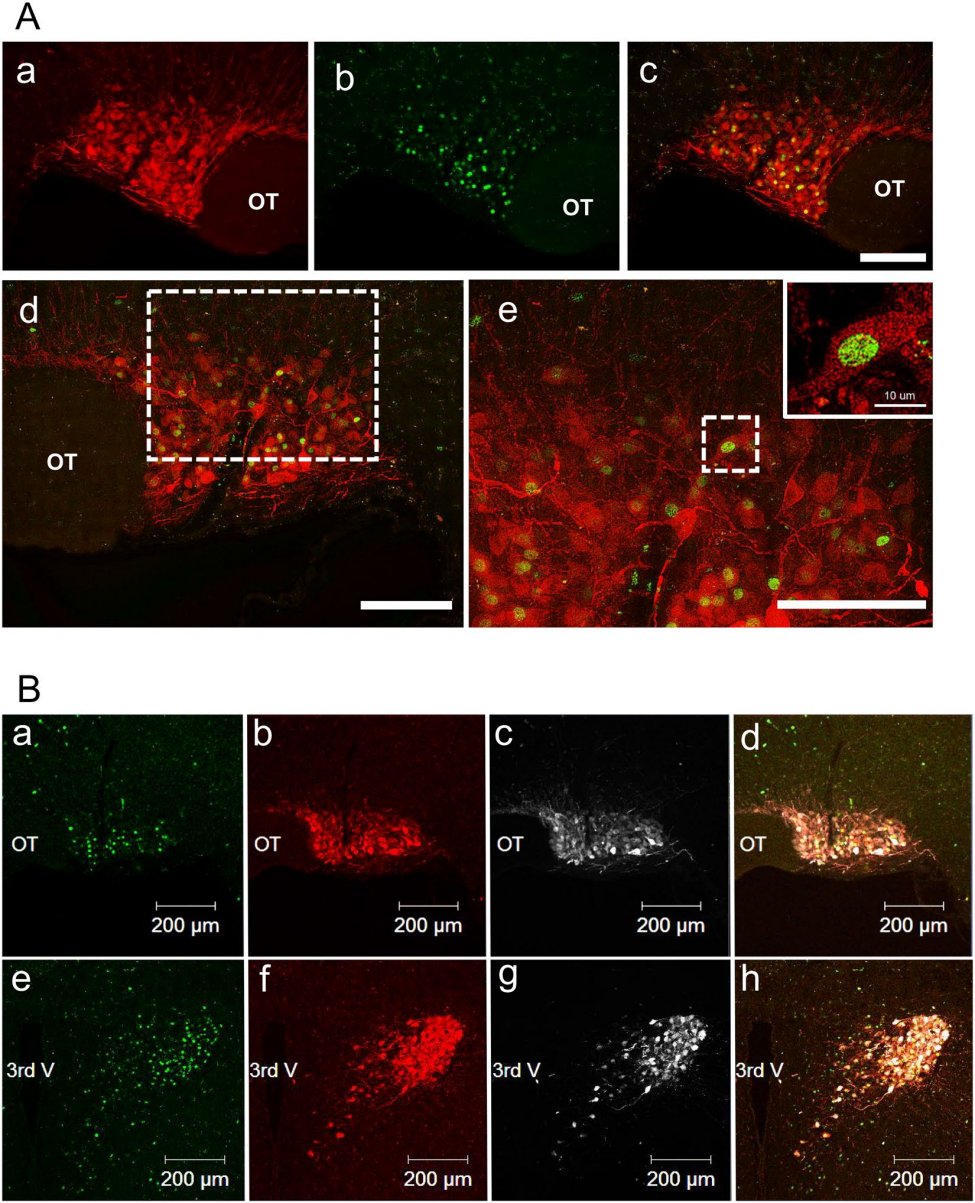
Expression of Fos-like immunoreactivity (LI), hM3Dq-mCherry fluorescence, and AVP-LI in the SON and PVN after i.p. administration of CNO. (**A**) Representative image of an hM3Dq-mCherry neuron expressing Fos in the SON 90 min after intraperitoneal (i.p.) administration of clozapine-N-oxide (CNO, 1 mg/kg). The mCherry fluorescence positive neuron (a), Fos-like-immunoreactive (-LI) neuron (b), and merged image (c) are shown, respectively. The surrounded white dotted line in merged image in the SON (d) is enlarged in panel e (e). Scale bars in low-power photomicrograph indicate 100 µm and high-power photomicrograph indicates 10 µm. OT, optic tract. (**B**) Digital images of double FIHC for Fos (a and e) and AVP (c and g) in the SON (a–d) and PVN (e–h) 90 min after i.p. administration of CNO (1 mg/kg) are shown. Endogenous hM3Dq-mCherry fluorescence was also observed (b and f). Merged image of a–c and e–g are demonstrated in d and h, respectively. Scale bars indicate 200 μm. OT, optic tract; 3^rd^ V, third ventricle.

To confirm that hM3Dq are expressed in the mCherry neurons and these GPCRs are functioning, and that mCherry neurons were specifically expressed in AVP neurons, we performed fluorescent immunohistochemistry (FIHC) for Fos and AVP 90 min after intraperitoneally (i.p.) administration of CNO (1 mg/kg). Fos-like-immunoreactivity (-LI) was induced in the hM3Dq-mCherry neurons in the SCN (Fig. 2A). mCherry neurons were expressed in all AVP neurons in the SON and PVN (Fig. 2B), suggesting that these hM3Dq are functioning.

**Figure 3:**
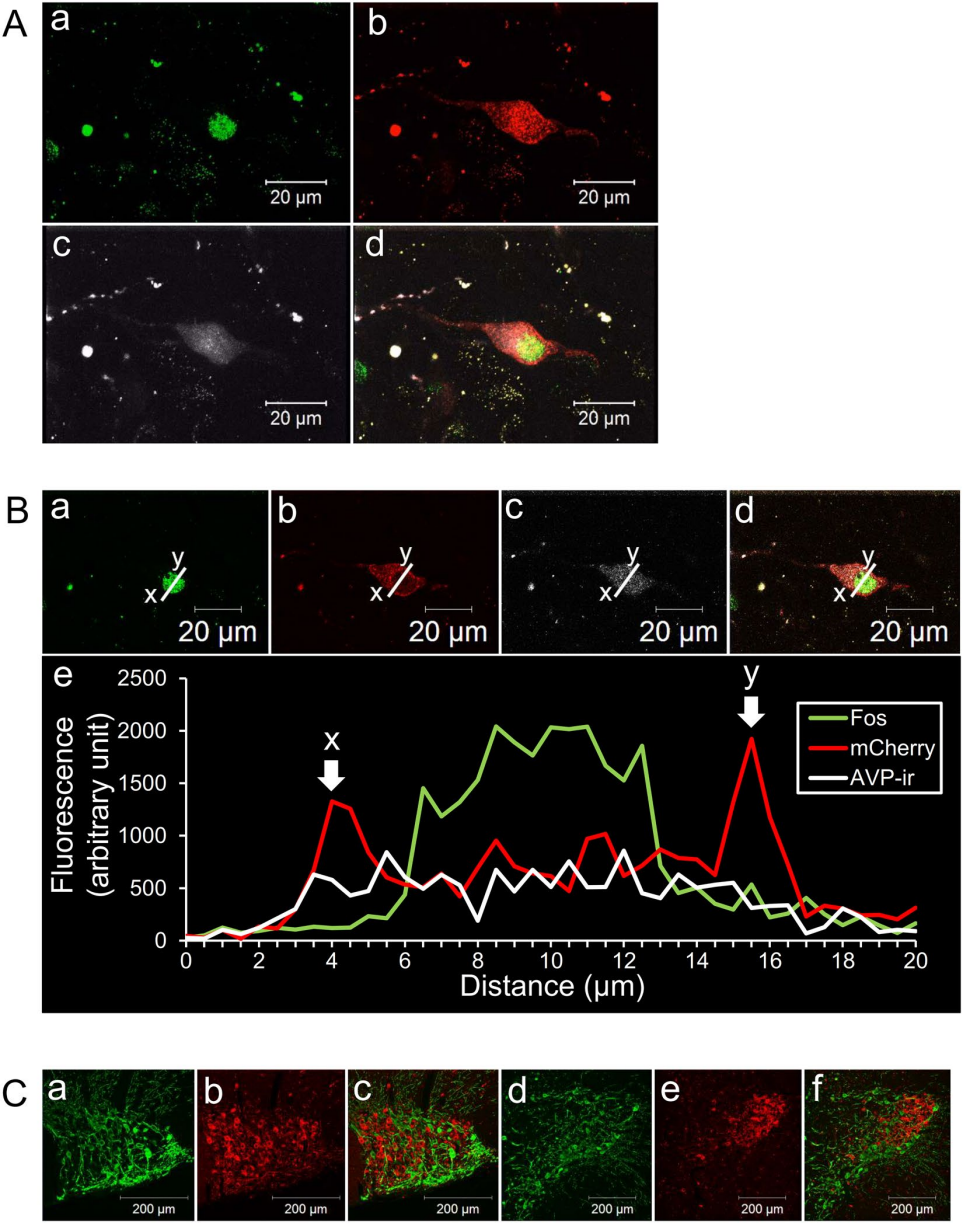
Fluorescent intensity profile and FIHC for OXT. (**A**) Reconstructed 3D images of an AVP neuron in the PVN 90 min after i.p. administration of CNO (1 mg/kg) are shown. Fos-LI was observed as green round-shaped nucleus (a). Endogenous hM3Dq-mCherry fluorescence seemed to be expressed mainly around the membrane of a neuron and dendrite (b), whereas AVP-LI is expressed diffusely in the cytoplasm (c). Merged image of a-c is shown in panel d (d). Scale bars indicate 20 μm. (**B**) Fluorescent intensity was analised in cross section by using confocal laser scanning microscope. The images were obtained from the AVP-hM3Dq-mCherry transgenic rats given CNO (1 mg/kg). Fos-LI was observed in the nucleus of an AVP neuron (a and e). Endogenous hM3Dq-mCherry was located mainly in the membrane (b and e) and AVP-LI was distributed diffusely in the cytoplasm (c and e). Merged image obtained from a-c is demonstrated in panel d (d). Scale bars indicate 20 μm. The fluorescence intensity profiles of Fos-LI (green), endogenous hM3Dq-mCherry (red), and AVP-LI (white) were measured at the location of straight line (x-y), respectively (e). (**C**) FIHC for OXT was performed in the AVP-hM3Dq-mCherry transgenic rat line. OXT-LI (a and c) and endogenous hM3Dq-mCherry (b and d) was observed in the SON (a–c) and PVN (d–f). Merged images of the OXT-LI and endogenous hM3Dq-mCherry in the SON (c) and PVN (f) are demonstrated. Scale bars indicate 200 μm.

Three dimensional reconstructed image of an AVP neuron looks like to be covered with mCherry fluorescence (Fig. 3Ab), whereas AVP-LI were not (Fig. 3Ac). Fos-LI was observed in the nucleus (Fig. 3Aa) 90 min after i.p administration of CNO (1 mg/kg). Confocal laser scanning microscopic observations of a cross section of an AVP neuron revealed that mCherry fluorescence were localised densely in the membrane of the PVN neurons (Fig. 3B). Some aggregated mCherry-positive spots were observed in the cytoplasm (Fig. 3Bb and e), however, their fluorescence intensity was lower when compared to the signal in the membrane. FIHC showed that endogenous AVP was diffusely distributed throughout the cytoplasm in mCherry neuron (Fig. 3Bc and e).

Under fluorescent microscopic observation, 3 out of 84 mCherry neurons in the SON (n = 4) (Fig. 3Ca) and 2 of 92 mCherry neurons in the PVN (n = 4) (Fig. 3Cb) were merged with OXT-LI neurons. These data indicate that less than 5% of mCherry neurons exhibited cytoplasmic OXT. This is consistent with previous study that 3–5% of MNCs expressed equivalent levels of AVP and OXT^10^.

### Percentage of Fos induction in mCherry neurons in the SON and PVN

**Figure 4:**
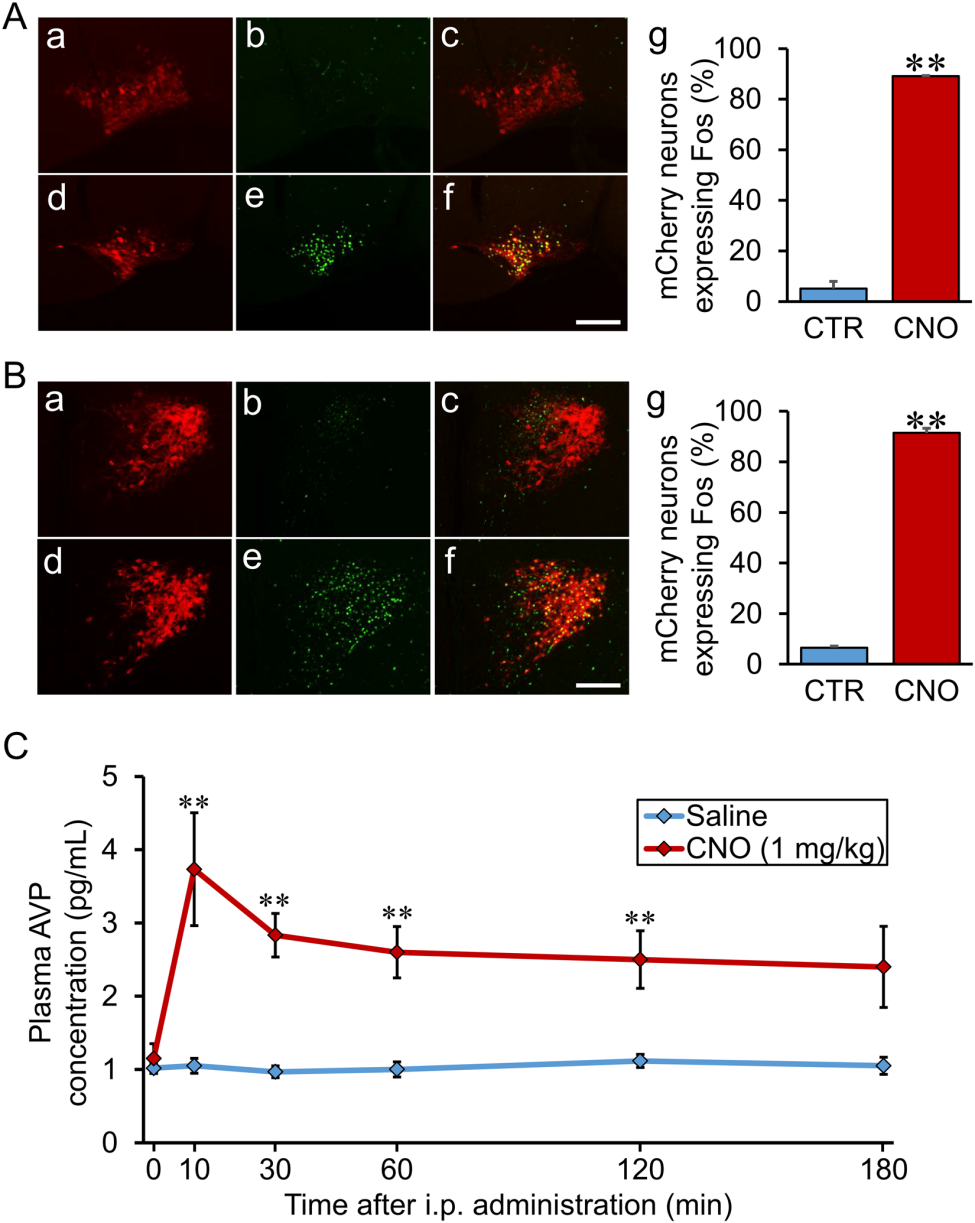
Percentage of Fos induction in mCherry neurons in the SON and PVN and plasma AVP concentration after i.p. administration of CNO. (**A**) Digital images of endogenous hM3Dq-mCherry neurons (a and d), Fos-LI neurons (b and e), and their merged images (c and f) of the SON are displayed. The group given saline as control (CTR) (a–c) or CNO (1 mg/kg) (d–f) were compared 90 min after i.p. administration of each compound. The percentages of hM3Dq-mCherry neurons expressing Fos-LI in the SON were counted manually (g). Scale bar indicates 100 μm. **P < 0.01 vs. CTR. Data are presented as means ± SEM (n = 6 each). (**B**) Digital images of endogenous hM3Dq mCherry neurons (a and d), Fos-LI neurons (b and e), and their merged images (c and f) of the PVN are displayed. The group given saline as control (CTR) (a-c) or CNO (1 mg/kg) (d-f) were compared 90 min after i.p. administration of each compound. The percentages of hM3Dq-mCherry neurons expressing Fos-LI in the PVN were counted manually (g). Scale bar indicates 100 μm. **P < 0.01 vs. CTR. Data are presented as means ± SEM (n = 6 each). (**C**) Plasma AVP concentration was measured at 0 min, 10 min, 30 min, 60 min, 120 min, and 180 min after i.p. administration of saline or CNO (1 mg/kg) (n = 4–6 in each group at each time point). **P < 0.01 vs. CTR. Data are presented as means ± SEM.

To examine what extent of hM3Dq in the SON and PVN are activated by i.p. administration of CNO, we counted the number of mCherry neurons expressing Fos by performing FIHC for Fos 90 min after i.p. administration of CNO (1 mg/kg). Significant induction of Fos was observed in the SON (89.1 ± 0.5%) and PVN (91.5 ± 3.9%) 90 min after i.p. administration of CNO (1 mg/kg) compared to control (SON; 5.1 ± 4.9%, PVN; 6.5 ± 1.1%) (Fig. 4A,B).

### Plasma AVP concentration

We measured plasma AVP concentration after i.p. administration of CNO (1 mg/kg). Plasma AVP concentration was significantly elevated at 10, 30, 60, and 120 min compared to saline administration (Fig. 4C). As plasma AVP concentration was increased just after i.p. administration of CNO, it is indicated that hM3Dq may be expressed not only in the cell body but also in the terminals (posterior pituitary) of AVP neurons.

### Food intake, water intake, and urine volume

**Figure 5:**
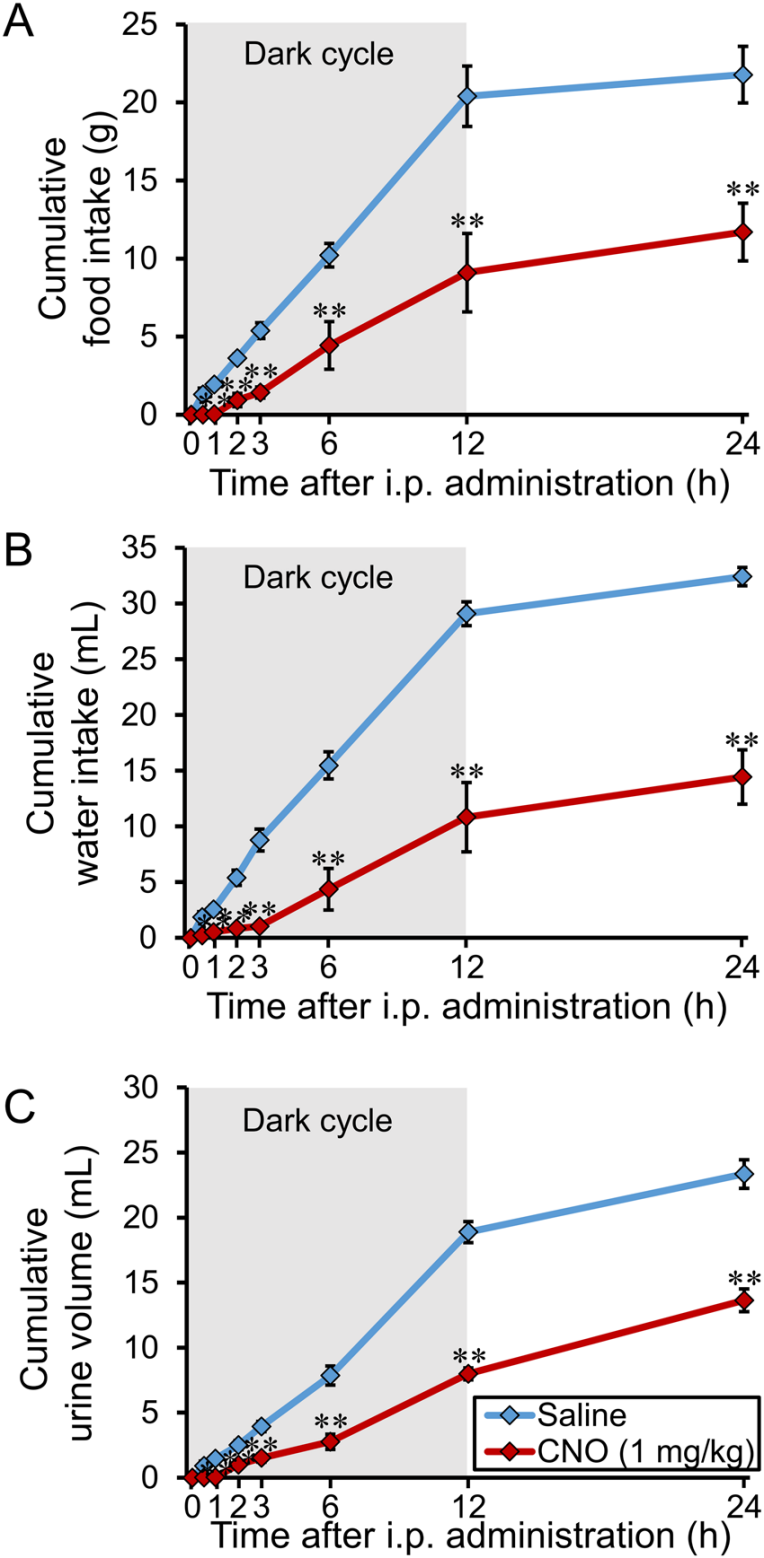
Cumulative food intake, water intake, and urine volume after i.p. administration of CNO. (**A**) Effects of i.p. administration of saline or CNO (1 mg/kg) on food intake in AVP-hM3Dq-mCherry transgenic rats at 0 h, 0.5 h, 1 h, 2 h, 3 h, 6 h, 12 h, and 24 h. Data are presented as means ± SEM (n = 6 each). **P < 0.01 vs. Saline. (**B**) Effects of i.p. administration of saline or CNO (1 mg/kg) on water intake in AVP-hM3Dq-mCherry transgenic rats at 0 h, 0.5 h, 1 h, 2 h, 3 h, 6 h, 12 h, and 24 h. Data are presented as means ± SEM (n = 6 each). **P < 0.01 vs. Saline. (**C**) Effects of i.p. administration of saline or CNO (1 mg/kg) on urine volume in AVP-hM3Dq-mCherry transgenic rats at 0 h, 0.5 h, 1 h, 2 h, 3 h, 6 h, 12 h, and 24 h. Data are presented as means ± SEM (n = 6 each). **P < 0.01 vs. Saline.

Food intake, water intake, and urine volume were significantly attenuated after i.p. treatment of CNO (1 mg/kg) at least for 24 h (Fig. 5A–C).

### Percentage of Fos induction in mCherry neurons in the SCN

**Figure 6:**
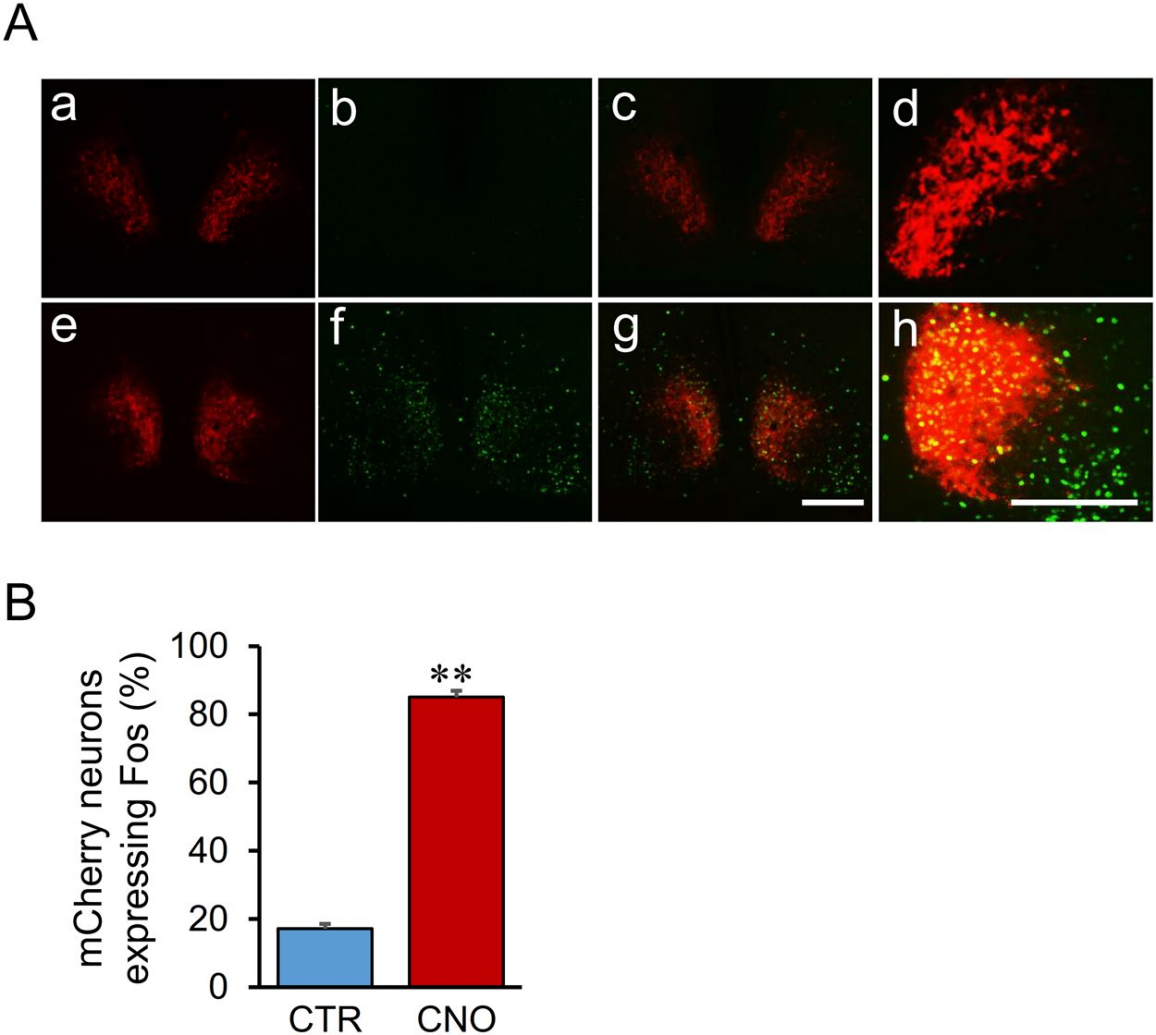
Percentage of Fos induction in mCherry neurons after i.p. administration of CNO in the SCN. (**A**) Digital images of endogenous hM3Dq mCherry neurons (a and d), Fos-LI neurons (b and e), and their merged images (c and f) of the SCN are displayed. The group given saline as control (CTR) (a-c) or CNO (1 mg/kg) (d–f) were compared 90 min after i.p. administration of each compound. Scale bar indicates 100 μm. (**B**) The percentages of hM3Dq-mCherry neurons expressing Fos-LI in the SCN were counted manually. **P < 0.01 vs. CTR. Data are presented as means ± SEM (n = 6 each).

To examine whether the decreased food intake was due, at least, to the activation of AVP neurons in the suprachiasmatic nucleus (SCN), we performed FIHC for Fos 90 min after i.p. administration of CNO. Significant induction of Fos was observed in the SCN (85.0 ± 4.2%) 90 min after i.p. administration of CNO (1 mg/kg) compared to control (17.1 ± 2.5%) (Fig. 6A and B).

## Discussion

We established a novel transgenic rat line that express hM3Dq and mCherry fluorescence specifically in AVP neurons. We demonstrated that the mCherry neurons were specific to AVP neurons in the hypothalamus that are known to contain AVP neurons. Fos induction was observed in these mCherry neurons after i.p. administration of CNO, which means that hM3Dq tagged with mCherry fluorescence are functioning. Results of plasma AVP concentration also suggested that these hM3Dq are functioning. AVP and OXT are intermingled in the hypothalamus. The stimuli, such as salt loading or osmotic stimuli, activate AVP and/or OXT neurons. In the latter half of 19^th^ century, it was found that milk ejection reflex or cholecystokinin (CCK) specifically activated OXT neurons^11,12^. These findings dramatically advanced the understanding of the properties of OXT neurons. However, the stimulation which specifically activates AVP neurons in the brain has not been discovered yet. Thus, it is expected that the property of AVP neurons would be progressed as in the examples of OXT, by using this transgenic rat line.

In general, viral vectors such as AAV/lenti virus, rather than transgenic approaches, have been used to express the hM3Dq in specific population of target cells^13,14^. We need to express GPCRs such as hM3Dq in the targeted neurons abundantly in order to evoke action potentials by a specific drug. To date, we have much experience in transgenic techniques which are relevant in the present context, including the generation of transgenic rat lines that express exogenous fusion genes such as AVP-eGFP^15^, OXT-mRFP1^16^, c-*fos*-mRFP1^17^, c-*fos*-eGFP^18^, and AVP-Channelrhodopsin 2-eGFP^19^. From our results of a novel transgenic rat line, it could be said that hM3Dq is expressed in AVP neurons sufficiently, which would be necessary for neuronal activation by administration of CNO. In addition, we confirmed that these transgenic fusion genes are definitely inherited to progenies. The excel points to use transgenic approaches are that the expression levels of hM3Dq-mCherry are stable in a line. We may be able to ignore the difference of gene expression levels as used in viral vectors injections. And by using transgenic technique, we can exclude the side effects of inflammatory processes both due to the virus and the microinjection itself.

As hM3Dq is one of the GPCRs, it is expected to be expressed in the membrane of a neuron. Our confocal microscopic observation revealed that hM3Dq-mCherry was densely localised to the membrane of an AVP neuron. We consider that mCherry-positive spots may be artifacts and/or non-functional clusters in the cytoplasm, or, these may be transported from nucleus to membrane.

Induction of Fos was observed in about 80 to 90% of the mCherry positive neurons in the SON, PVN, and SCN. The reason why all of the mCherry neurons were not activated after i.p. administration of CNO remains unclear. One possibility was due to the dosage of CNO administration because the dosage (1 mg/kg) was unified throughout the experiments in the present study. We should try another dosage to clarify the effects of CNO on AVP neurons by further study, however, there are some issues to be noticed. Although, CNO is thought to be largely an inactive metabolite^20,21^, some studies reported that administration of CNO caused detectable levels of clozapine which was retro-converted from CNO in the plasma of humans and guinea pigs^22,23^, which may affect animals behavior. Thus, we should pay close attention to the dosage of CNO when conducting behavioural experiments by using DREADDs system. Recently, Gomez *et al*. reported that it is possible that clozapine, but not CNO, activated hM3Dq in the brain^24^. We should try another compound like perlapine hydrochloride, which is a novel, potent and selective, non-CNO hM3Dq DREADD receptor agonist by further study.

It raises concerns that if this transgenic rat model silence AVP neuron when it is not activated by CNO or chemical stimuli is not specific to activate the transgene. However, these possibilities seem to be negative, because Fos induction were not significant between saline-treated and CNO treated wild type Wistar rats nor between saline-treated non-transgenic Wistar rats and transgenic rats (Suppl. Figure 1). In addition, plasma AVP levels (Suppl. Figure 2), and behavioural changes (Suppl. Figure 3) were comparable after i.p. administration of CNO in non-transgenic Wistar rats. Thus, chemical stimuli of CNO may almost be ignored.

Urine volume was significantly decreased after i.p. administration of CNO. According to our results, plasma AVP was markedly elevated at least for 120 min after i.p. administration of CNO. This seems to be reasonable as plasma AVP concentration was significantly increased. Decreased water intake may be due to the reduction of the urine volume. We demonstrated, for the first time, that endogenous AVP activation decreased food intake by using this transgenic rat line. One possible explanation of this bahavioural change was due to the disruption of the circadian rhythmicity, as AVP neurons in the SCN, as well as SON and PVN, was dramatically activated by CNO administration. Locomotor activity and body temperature were disturbed after i.p. administration of CNO for 2 or 3 days (Suppl. Figure 4). These data may support our hypothesis. Lots of unveiled mechanism regarding attenuated food intake should be elucidated by further study, e.g. other feeding regulating neuropeptides would be activated under the downstream of the central AVP signaling. However, in the present study, we put emphasis on reporting the immediacy of generating this novel transgenic rat line.

Although DREADDs technique is noninvasive as it uses pharmacological methods, the temporal resolution is one of the disadvantages for investigating acute behaviours^3^. We consider this novel transgenic rat line as a powerful animal model for behavioral experiments depending upon longer-lasting alterations. In addition, we should keep note that G protein coupled signaling is not as simple as ion channels and diverse thing would be altered. We should conduct electrophysiology to reveal how AVP neurons respond to activation by CNO because some AVP neurons expressing hM3Dq may induce long-lasting spikes, whilst other AVP neurons may encounter periodic depolarization blocks^9,25^.

As a next step, we should confirm whether hM3Dq-mCherry also localises to the dendrites and axons, and whether AVP release from the axon terminals could be triggered by administration of CNO. Although the detailed mechanism has unveiled, we demonstrated, for the first time, that the activation of endogenous AVP neurons after CNO administration decreased food intake, by using AVP-hM3Dq-mCherry transgenic rat line. This novel transgenic rat line may provide a revolutionary insight into the neuronal mechanism regarding central AVP system responsible for various kind of behaviours.

## Materials and Methods

### Animals and Ethical Approval

Non-transgenic and heterozygous transgenic Wistar (CrLj:WI, Japan Charles River, Yokohama, Japan) rats were bred and housed under normal laboratory conditions (12 h light, 12 h dark cycle, lights on at 07.00 h) with free access to food and tap water. All experiments in this study were performed in strict accordance with guidelines on the use and care of laboratory animals’ asset out by the Physiological Society of Japan and approved by the Ethics Committee of Animal Care and Experimentation, University of Occupational and Environmental Health.

### Constructs for microinjection

A chimeric AVP-hM3Dq-mCherry BAC clone transgene construct was purified for microinjections. The hM3Dq-mCherry sequence from the hM3Dq-mCherry cassette (Plasmid #44361, Addgene, Cambridge, MA, USA) was used for the transgene. Detailed procedures were described previously^16,26^. Next, SV40 poly A sequence was framed to the hM3Dq-mCherry sequence. Finally, this hM3Dq-mCherry-SV40 poly A cassette was introduced into the rat AVP gene in place of the genomic start codon. Thus, hM3Dq-mCherry should be specifically expressed under the AVP promoter in the transgenic rat brain. Transgenic founders and germ line transmission of the BAC transgene construct was assessed by Southern blotting, using ^32^P-labeled mCherry probes. Transgenic founder rats were bred with non-transgenic Wistar rats. F1 heterozygous transgenic rats were screened by PCR analysis of genomic DNA extracted from ear skin as previously described^15^.

### Expression of hM3Dq-mCherry in AVP neurons

The rats were deeply anesthetized with intraperitoneal injection of cocktail of three different anaesthetic agents (in combination with 0.3 mg/kg of medetomidine, 4.0 mg/kg of midazolam, and 5.0 mg/kg of butorphanol). They were perfused transcardially with 0.1 M phosphate buffer (PB) (pH 7.4) containing heparin (1000 U/l), followed by 4% paraformaldehyde in 0.1 M PB. The brains were carefully removed, and a small block that included the hypothalamus was isolated. The blocks were post-fixed with 4% paraformaldehyde in 0.1 MPB for 48 h at 4 °C as previously^15,17^. The tissue was cryoprotected in 20%-(w/v) sucrose in 0.1 M PB for 48 h at 4 °C. Fixed tissue was cut at 40 μm by using a microtome (REM-700; Yamato Kohki Industrial Co. Ltd, Saitama, Japan). The sections were rinsed twice with 0.1 M PB and placed on glass slides. The sections, containing the SCN, SON, and PVN, were examined by fluorescent microscopy (ECLIPSEE 600; Nikon Corp., Tokyo, Japan) with an RFP filter (Nikon Corp.) to visualise hM3Dq-mCherry expression. The images were captured with a digital camera (DS-L2, DS-Fi1; Nikon Corp.). The observed nuclei were determined according to the coordinate that given in the rat brain atlas^27^.

### FIHC for Fos, AVP, and OXT

CNO (Sigma-Aldrich Japan Co. LLC., Tokyo, Japan) was dissolved in saline (Otsuka Pharmaceutical Co. LTD., Tokyo, Japan). Dosage of CNO was determined as described previously^28,29^. At 90 min after i.p. administration of saline (1 mL/100 g body weight, n = 6) or CNO (0.1 mg/mL/100 g body weight, n = 6), rats were deeply anaesthetized with three types of mixed anesthetic agents (in combination with 0.3 mg/kg of medetomidine, 4.0 mg/kg of midazolam, and 5.0 mg/kg of butorphanol). Detailed perfusion protocol is described above. Serial sections of 40 μm thickness were sliced using a microtome (REM-700, Yamato Kohki Industrial co. ltd., Tokyo, Japan). Sections were rinsed twice with 0.1 M phosphate-buffered saline (PBS) and washed in 0.1 M Tris buffer (pH 7.6) containing 0.3% Triton X-100.

For FIHC for Fos, sections were incubated for 48 h at 4 °C in a primary antibody solution (anti-c-Fos antibody raised in goat, Santa Cruz Biotechnology, TX, USA; 1:500). After washing twice in 0.3% Triton X-100 in PBS, floating sections were incubated for 2 h at 4 °C with a secondary antibody (Alexa Fluor 488 anti-goat IgG antibody raised in donkey; Molecular Probes, OR, USA; 1:2,000 in PBS containing 0.3% Triton X-100).

For FIHC for AVP, sections were incubated for 48 h at 4 °C in a primary antibody solution (anti-AVP antibody raised in rabbit, Lot.1004001, Immunostar Inc., WI, USA; 1:10,000). After washing twice in 0.3% Triton X-100 in PBS, floating sections were incubated for 2 h at 4 °C with a secondary antibody (Alexa Fluor 633 anti-rabbit IgG antibody raised in goat; Molecular Probes, OR, USA; 1:2,000 in PBS containing 0.3% Triton X-100).

For FIHC for OXT, sections were incubated for 48 h at 4 °C in a primary antibody solution (anti-OXT antibody raised in rabbit, AB911, Merck Corp. Darmstadt, Germany; 1:10,000). After washing twice in 0.3% Triton X-100 in PBS, floating sections were incubated for 2 h at 4 °C with a secondary antibody (Alexa Fluor 488 anti-rabbit IgG antibody raised in goat; Molecular Probes, OR, USA; 1:2,000 in PBS containing 0.3% Triton X-100).

They were then washed twice in PBS for 10 min, and mounted on the slide glass and cover-slipped by using a vectashield (Vector Laboratories Co. Ltd., CA, USA). Images scanned by confocal laser scanning microscopy were reconstructed and the fluorescence-intensity profile was measured at z-slices by using imaging software provided with the LSM5 PASCAL laser scanning microscope (Carl Zeiss Co. Ltd. Oberkochen, Germany).

### Percentage of Fos induction in mCherry neurons

Each captured microphotograph was printed onto a paper in expanded size. Then, the printed papers were blinded and Fos-positive (appearing as round-shaped and green-coloured), hM3Dq-mCherry-positive (appearing as red cytoplasmic cells) and double-positive cells (appearing as red cytoplasmic cells containing green nuclei in merged images) were counted by at least two researchers to avoid bias. We counted four cross sections (eight nuclei including right and left) of the each nucleus and the results were averaged. To prevent double-counting, we checked the cross mark in the printed paper every time we counted the number of Fos-positive, hM3Dq-mCherry-positive, and double-positive cells.

### Plasma AVP concentration

At 0 min, 10 min, 30 min, 60 min, 120 min, and 180 min after i.p. administration of saline or CNO (1 mg/kg), the rats were decapitated immediately without being anesthetized. The number of rats used in this experiment was 4–6 in each group at each time point. Trunk blood samples were collected into chilled reaction tubes (Greiner Bio-One Co. Ltd., Kremsmuenster, Austria) containing an aprotinin/EDTA mixture. Blood samples were centrifuged for 10 minutes at 4 °C for 1,000 *g*. After centrifuged, a 500 μL sample of plasma was taken for measuring plasma AVP concentration (SRL, Tokyo, Japan).

### Measurement of food intake, water intake, and urine volume

All rats were handled for 5 days before the experiment. They were housed in the metallic metabolic cages 2 days before the experiment to accustom the circumstances. At 19.00, just before the start of a dark cycle, saline (n = 6) or CNO (1 mg/kg, n = 6) were intraperitoneally administered, then the measurements were commenced. All rats had free access to food and water after the start of the measurements. Food intake, water intake, and urine volume were measured at 0 h, 0.5 h, 1 h, 2 h, 3 h, 6 h, 12 h, and 24 h after i.p. administration.

### Statistical analysis

The mean ± SEM was calculated from the results of the cumulative food intake, water intake, and urine volume and quantification of the FIHC studies. All data were analyzed by one-way ANOVA followed by a Bonferroni-type adjustment for multiple comparisons (Origin Pro version 8.5 J, Lightstone, Tokyo, Japan). Statistical significance was set at *P* < 0.05.

## Electronic supplementary material

Supplementary information

## References

[1] Deisseroth K (2011). Optogenetics. Nat Meth.

[2] English JG, Roth BL (2015). Chemogenetics-A Transformational and Translational Platform. JAMA Neurol..

[3] Smith KS, Bucci DJ, Luikart BW, Mahler SV (2016). DREADDs: Use and Application in Behavioral Neuroscience. Behav. Neurosci..

[4] Garrott K (2017). Chronic activation of hypothalamic oxytocin neurons improves cardiac function during left ventricular hypertrophy-induced heart failure. Cardiovasc. Res..

[5] Pei H, Sutton AK, Burnett KH, Fuller PM, Olson DP (2014). AVP neurons in the paraventricular nucleus of the hypothalamus regulate feeding. Mol. Metab..

[6] Smith AS, Williams Avram SK, Cymerblit-Sabba A, Song J, Young WS (2016). Targeted activation of the hippocampal CA2 area strongly enhances social memory. Mol. Psychiatry.

[7] Bendesky A (2017). The genetic basis of parental care evolution in monogamous mice. Nature.

[8] Brownstein MJ, Russell JT, Gainer H (1980). Synthesis, transport, and release of posterior pituitary hormones. Science (80-.)..

[9] Ludwig M, Leng G (2006). Dendritic peptide release and peptide-dependent behaviours. Nat. Rev. Neurosci..

[10] Kiyama H, Emson PC (1990). Evidence for the Co‐Expression of Oxytocin and Vasopressin Messenger Ribonucleic Acids in Magnocellular Neurosecretory Cells: Simultaneous Demonstration of Two Neurohypophysin Messenger Ribonucleic Acids by Hybridization Histochemistry. J. Neuroendocrinol..

[11] Higuchi T, Honda K, Fukuoka T, Negoro H, Wakabayashi K (1985). Release of oxytocin during suckling and parturition in the rat. J. Endocrinol..

[12] Verbalis JG, McCann MJ, McHale CM, Stricker EM (1986). Oxytocin secretion in response to cholecystokinin and food: differentiation of nausea from satiety. Science (80-.)..

[13] Knobloch HS (2012). Evoked axonal oxytocin release in the central amygdala attenuates fear response. Neuron.

[14] Choe HK (2015). Oxytocin Mediates Entrainment of Sensory Stimuli to Social Cues of Opposing Valence. Neuron.

[15] Fujio T (2006). Exaggerated response of arginine vasopressin-enhanced green fluorescent protein fusion gene to salt loading without disturbance of body fluid homeostasis in rats. J. Neuroendocrinol..

[16] Katoh A (2011). Highly visible expression of an oxytocin-monomeric red fluorescent protein 1 fusion gene in the hypothalamus and posterior pituitary of transgenic rats. Endocrinology.

[17] Yoshimura, M. *et al*. A c-fos-Monomeric Red Fluorescent Protein 1 Fusion Transgene is Differentially Expressed in Rat Forebrain and Brainstem after Chronic Dehydration and Rehydration. *J*. *Neuroendocrinol*. **25**, (2013). 10.1111/jne.12022 23350545

[18] Katoh, A. *et al*. Fluorescent visualisation of the hypothalamic oxytocin neurones activated by cholecystokinin-8 in rats expressing c-fos-enhanced green fluorescent protein and oxytocin-monomeric red fluorescent protein 1 fusion transgenes. *J*. *Neuroendocrinol*. **26**, (2014). 10.1111/jne.12150 24730419

[19] Ishii, M. *et al*. Transgenic approach to express the channelrhodopsin 2 gene in arginine vasopressin neurons of rats. *Neurosci*. *Lett*. **630**, (2016). 10.1016/j.neulet.2016.08.001 27493075

[20] Alves-Rodrigues A, Leurs R, Willems E, Timmerman H (1996). Binding of clozapine metabolites and analogues to the histamine H3 receptor in rat brain cortex. Arch. Pharm. (Weinheim)..

[21] Salmi P, Ahlenius S (1996). Further evidence for clozapine as a dopamine D1 receptor agonist. Eur J Pharmacol.

[22] Jann MW, Lam YW, Chang WH (1994). Rapid formation of clozapine in guinea-pigs and man following clozapine-N-oxide administration. Arch. Int. Pharmacodyn. thérapie.

[23] Chang WH (1998). Reversible metabolism of clozapine and clozapine N-oxide in schizophrenic patients. Prog. Neuro-Psychopharmacology Biol. Psychiatry.

[24] Gomez JL (2017). Chemogenetics revealed: DREADD occupancy and activation via converted clozapine. Science (80-.)..

[25] Brown CH, Ruan M, Scott V, Tobin VA, Ludwig M (2008). Multi-factorial somato-dendritic regulation of phasic spike discharge in vasopressin neurons. Progress in Brain Research.

[26] Fujihara H (2009). Robust up-regulation of nuclear red fluorescent-tagged fos marks neuronal activation in green fluorescent vasopressin neurons after osmotic stimulation in a double-transgenic rat. Endocrinology.

[27] Paxinos, G. & Watson, C. *The Rat Brain in Stereotaxic Coordinates Fourth Edition*. *Academic press* 0125476191 (1998).

[28] MacLaren, D. A. A. *et al*. Clozapine N-Oxide Administration Produces Behavioral Effects in Long-Evans Rats: Implications for Designing DREADD Experiments. *eNeuro* **3** (2016). 10.1523/ENEURO.0219-16.2016 PMC5089539 27822508

[29] Jaiswal PB, English AW (2017). Chemogenetic enhancement of functional recovery after a sciatic nerve injury. Eur. J. Neurosci..

