# Supplementary material for: Activation of endogenous arginine vasopressin neurons inhibit food intake: by using a novel transgenic rat line with DREADDs system
Source: Sci Rep. 2017 Nov 16;7:15728. doi: 10.1038/s41598-017-16049-2 (PMC5691068; doi:10.1038/s41598-017-16049-2)
Supplement: Supplementary file 1 — Supplementary information [file 41598_2017_16049_MOESM1_ESM.pdf]

Activation of endogenous arginine vasopressin neurons inhibit food intake: by using a novel transgenic rat line with DREADDs system

Abbreviated title: Endogenous vasopressin inhibit food intake

Mitsuhiro Yoshimura[1], Kazuaki Nishimura[1], Haruki Nishimura[2], Satomi Sonoda[1], Hiromichi Ueno[1], Yasuhito Mitojima[1, 2], Reiko Saito[1], Takashi Maruyama[1], Yuki Nonaka[1], and Yoichi Ueta[1]

[1]Department of Physiology, [2]Orthopedic surgery, School of Medicine, University of Occupational and Environmental Health, Kitakyushu 807-8555, Japan

Correspondence to: yoichi@med.uoeh-u.ac.jp (Yoichi Ueta)

Department of Physiology, School of Medicine, University of Occupational and Environmental Health, 1-1 Iseigaoka, Yahatanishi-ku, Kitakyushu 807-8555, Japan.

Tel.: +81-93-691-7420, Fax: +81-93-692-1711

Supple. Fig. 1

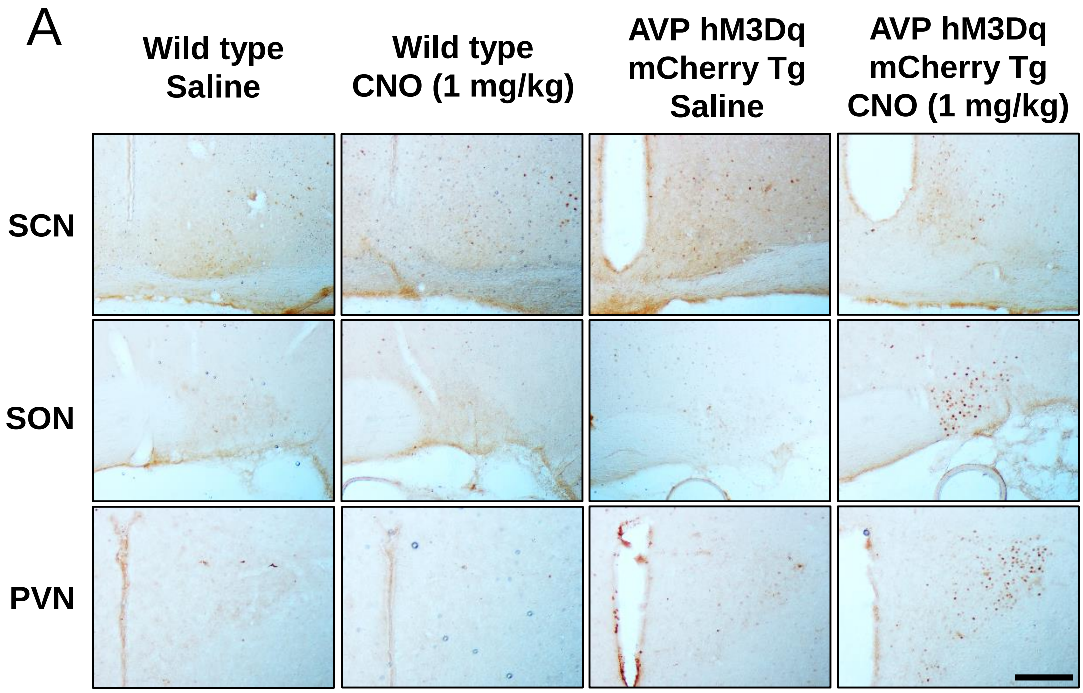

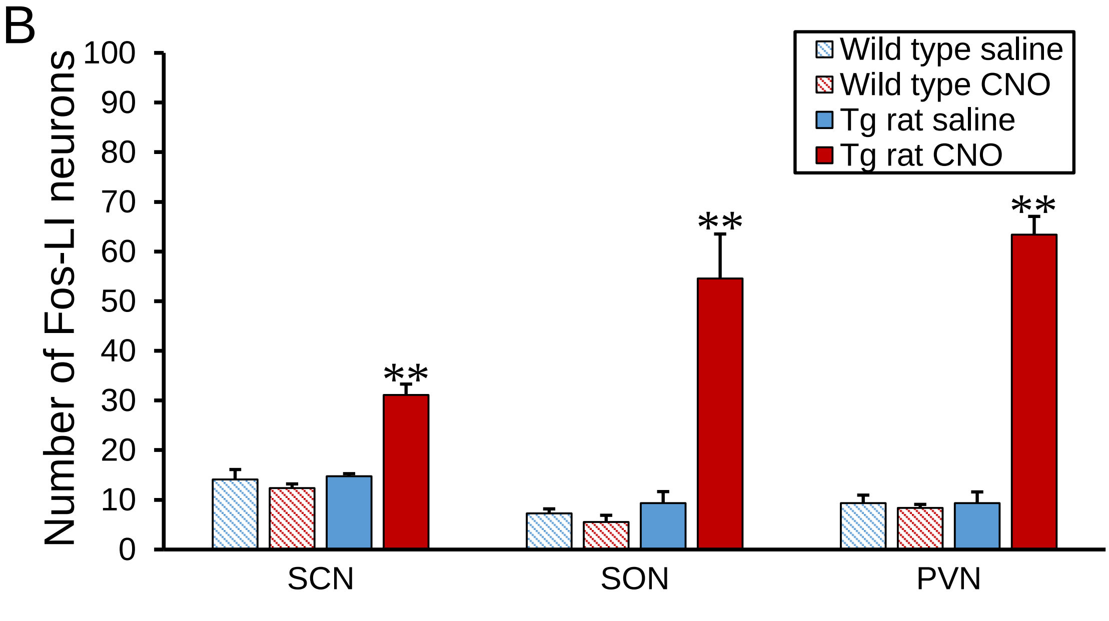

Supple. Fig. 1 Fos induction was not observed after i.p. administration of CNO in wild type rats.

(A) Digital images of Fos-LI in the SCN, SON, and PVN which were obtained from 90 min after i.p. administered saline or CNO (1 mg/kg) in adult male non-transgenic Wister rats and AVP-hM3Dq-mCherry transgenic rats (n=3 each). Scale bar indicate 200 μm.

(B) Quantitative analysis of number of Fos-LI neurons in the SCN, SON, and PVN. Fos-LI neurons were manually counted in three cross sections (six nuclei including right and left) of the each nucleus and the results were averaged. **P<0.01 vs. all other groups. Data are presented as mean ± SEM (n=3 each).

Supple. Fig. 2

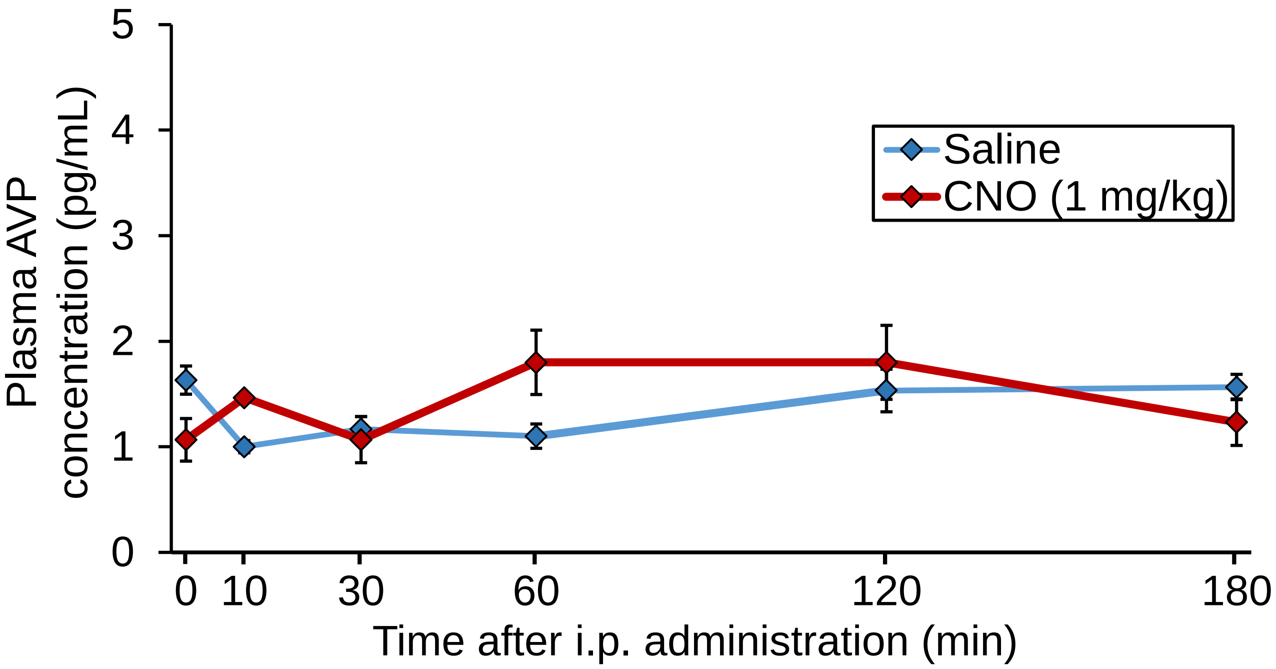

Supple. Fig. 2 Plasma AVP concentration after i.p. administration of CNO in wild type rats.

Plasma AVP concentration were comparable for 180 min after i.p. administration of saline or CNO (1 mg/kg) in adult male non-transgenic Wistar rats. Data are presented as mean ± SEM (n=3 in each group at each time point).

Supple. Fig. 3

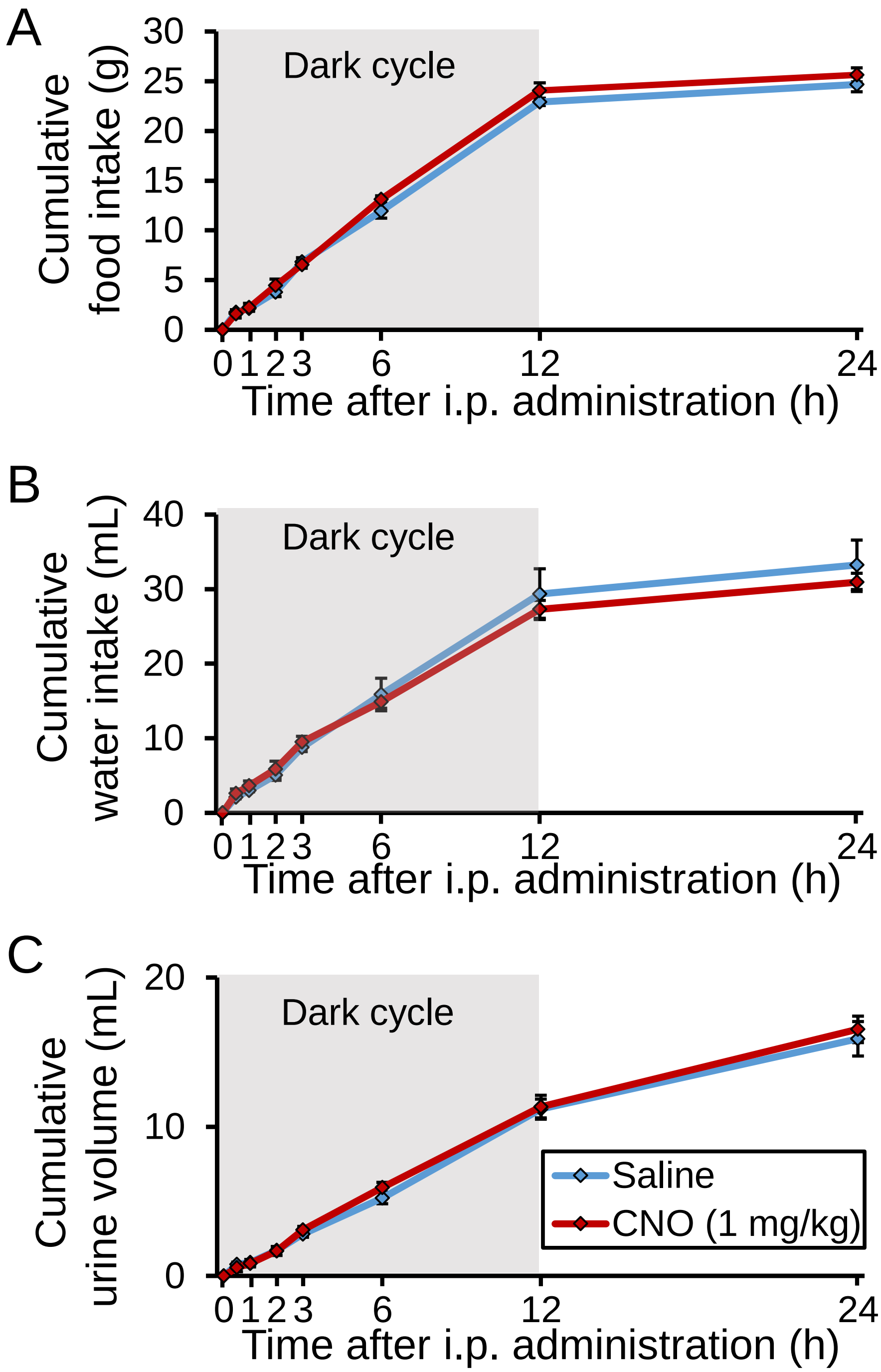

Supple. Fig. 3 I.p. administration of CNO did not affect food intake, water intake, nor urine volume in wild type rats.
Saline or CNO (1 mg/kg) was i.p. administered at 19:00 (start of a dark cycle) in adult male non-transgenic Wistar rats (180-210 g). Cumulative food intake (A), water intake (B), and urine volume (C) were comparable between saline group and CNO group for 24 h. Data are presented as mean ± SEM (n=5-6 each).

Supple. Fig. 4

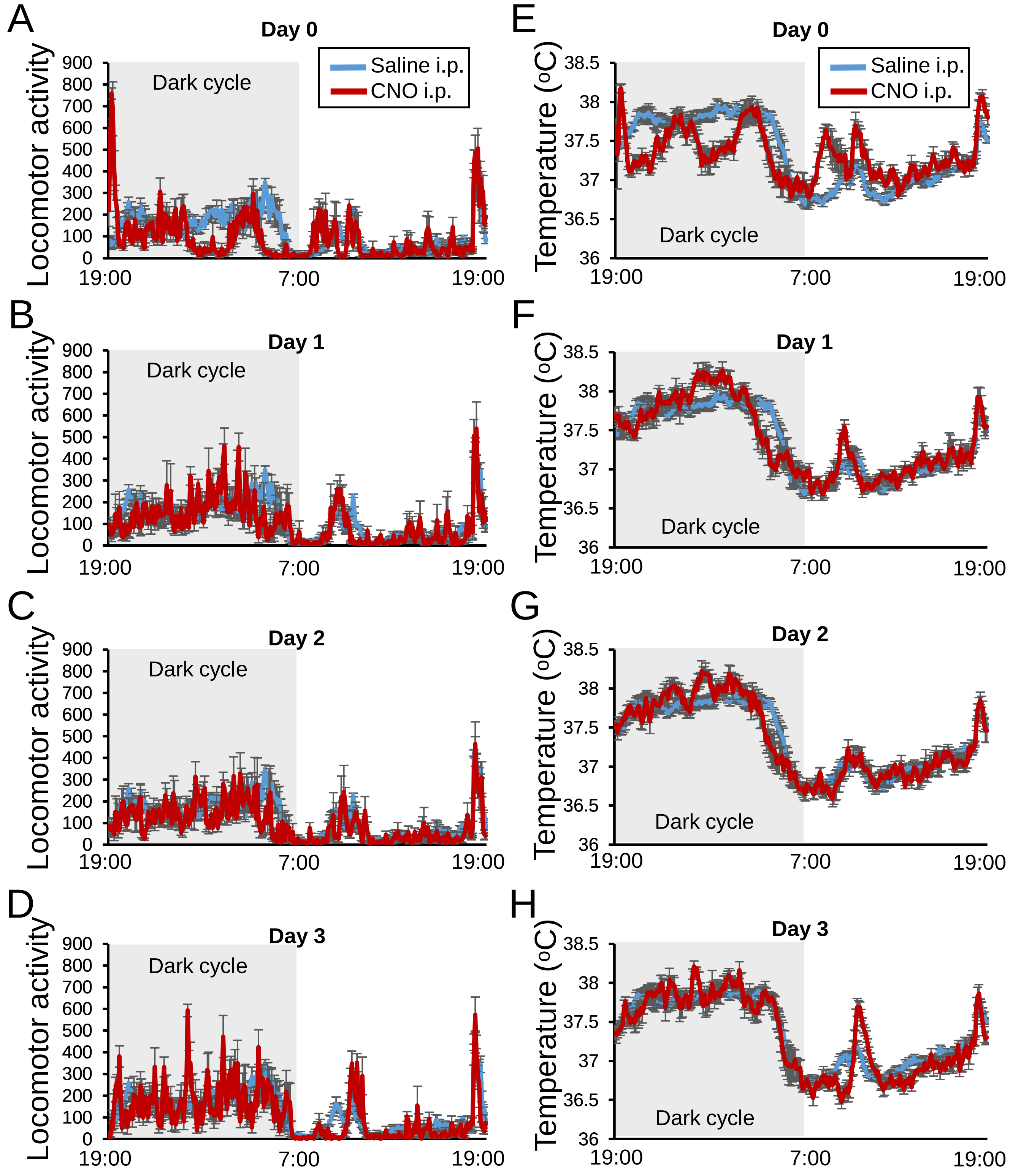

Supple. Fig. 4 Locomotor activity and body temperature after i.p. administration of CNO in AVP-hM3Dq-mCherry transgenic rats.

Nano tag (KISSEI COMTEC, Japan) was intraperitoneally implanted 2 weeks before the experiment. Saline or CNO (1 mg/kg) was i.p. administered only one time at 19:00 (start of a dark cycle) at day 0. Panel (A) to (D) indicate locomotor activity and panel (E) to (H) indicate body temperature. Locomotor activity and body temperature were disturbed after endogenous AVP activation at day 0 (A and E), day 1 (B and F), and day 2 (C and G). They turned back to baseline at day 3 (D and H). Data are presented as mean ± SEM (n=5 each).
